# Factors Predicting Discordant Virological and Immunological Responses to Antiretroviral Therapy in HIV-1 Clade C Infected Zulu/Xhosa in South Africa

**DOI:** 10.1371/journal.pone.0031161

**Published:** 2012-02-14

**Authors:** Boris Julg, Danielle Poole, Musie Ghebremichael, Carmen Castilla, Marcus Altfeld, Henry Sunpath, Richard A. Murphy, Bruce D. Walker

**Affiliations:** 1 Ragon Institute of MGH, MIT and Harvard, Boston, Massachusetts, United States of America; 2 HIV Pathogenesis Programme, Doris Duke Medical Research Institute and KwaZulu Natal Research Institute for TB and HIV, University of KwaZulu Natal, Durban, South Africa; 3 Department of Biostatistics and Computational Biology, Harvard University and Dana Farber Cancer Institute, Boston, Massachusetts, United States of America; 4 University of Medicine and Dentistry of New Jersey, Newark, New Jersey, United States of America; 5 McCord Hospital, Durban, South Africa; 6 Operational Support Unit, Doctors Without Borders, New York, New York, United States of America; 7 Howard Hughes Medical Institute, Chevy Chase, Maryland, United States of America; University of Hawaii, United States of America

## Abstract

Factors predicting suboptimal CD4 cell recovery have been studied in HIV clade-B infected US and European populations. It is, however, uncertain to what extent these results are applicable to HIV clade-C infected African populations. Multivariate analysis using logistic regression and longitudinal analyses using mixed models were employed to assess the impact of age, gender, baseline CD4 cell count, hemoglobin, body mass index (BMI), tuberculosis and other opportunistic co-infections, and frequencies of regimen change on CD4 cell recovery at 12 and 30 months and on overtime change in CD4 cells among 442 virologically suppressed South Africans. Despite adequate virological response 37% (95% CI:32%–42%) and 83% (95% CI:79%–86%) of patients on antiretroviral therapy failed to restore CD4 cell counts ≥200 cells/mm^3^ after 12 and ≥500 cells/mm^3^ after 30 months, respectively, in this South African cohort. Critical risk factors for inadequate recovery were older age (p = 0.001) and nadir CD4 cell count at ART initiation (p<0.0001), while concurrent TB co-infection, BMI, baseline hemoglobin, gender and antiretroviral regimen were not significant risk factors. These data suggest that greater efforts are needed to identify and treat HAART-eligible patients prior to severe CD4 cell decline or achievement of advanced age.

## Introduction

The roll-out of combination antiretroviral therapy has reduced AIDS-related morbidity and mortality in South Africa [1], [2], [3], [4]. Despite the potency of HAART, it has been observed that a significant proportion of patients – despite an adequate virological response – do not achieve CD4 cell recovery above critical thresholds associated with opportunistic infections. The need to better understand factors influencing CD4 cell restoration was underscored by a study from Cape Town, South Africa which found that CD4 cell count was the variable most strongly associated with mortality risk during HAART in South Africa and that a high cumulative mortality risk was associated with person-time accrued at low CD4 cell counts [2].

Although factors affecting CD4 cell restoration have been studied in Western cohorts it is uncertain to what extent these findings are applicable to African populations with HIV-1 clade C virus and distinct demographic as well as epidemiological characteristics [5], [6], [7], [8], [9], [10], [11], [12]. In this study we retrospectively examined CD4 cell count trajectories of 442 HIV-1 clade-C infected Zulu/Xhosa in KwaZulu-Natal Province, South Africa, who presented with CD4 cell counts <200 cells/mm^3^ at HAART initiation. Using mixed and logistic regression models we assessed the predictive value of baseline patient factors, incl. age, gender, hemoglobin, body-mass-index (BMI), baseline CD4 cell counts, presence of opportunistic infections (OI) and antiretroviral therapy regimen on overtime change in CD4 cell counts and CD4 cell restoration at 12 and 30 months.

## Methods

### Patients

We included patients ≥18 years of age with baseline CD4 cell counts of <200 cells/ml, who initiated their first HAART regimen in 2005 and 2006 at McCord Hospital, KwaZulu-Natal Province, South Africa and who achieved viral suppression, defined as having had at least 2 consecutive plasma HIV RNA levels <50 copies/mL within the first 48 weeks of therapy. HAART was defined as treatment with ≥3 antiretroviral drugs, including two nucleoside reverse-transcriptase inhibitors plus a non-nucleoside reverse-transcriptase inhibitor or a protease inhibitor. Observations after the study baseline were censored when the plasma HIV RNA level increased above 1000 copies/mL on two occasions, as this was considered to be treatment failure. Patients were also censored if they became lost to follow-up, died or if they had no plasma HIV RNA testing performed for >1 year. Patients who modified their HAART regimens were not censored when plasma HIV RNA levels remained <50 copies/mL. The McCord Research Ethics Committee, McCord Hospital, Durban, South Africa and the Partners Human Research Committee, Massachusetts General Hospital, Boston, USA both granted ethical approval for this study. As reviewed by the IRB the clinical data recorded in this study was considered non-identifiable (research code with no link), therefore it was determined consent was not required.

### Study Measures

Socio-demographic characteristics (including gender and age), antiretroviral treatment information, CD4 cell counts (at 0, 6, 12, 24 and 30 months post treatment initiation), HIV plasma RNA levels (at 6, 12, 24 and 30 months post treatment initiation), hemoglobin (Hgb) level and body-mass-index (BMI) at time of HAART initiation were collected from computerized clinic records and patient charts. In addition, clinical information regarding the presence of *Mycobacterium tuberculosis* (TB) infection (pulmonary, extra-pulmonary and TB meningitis) defined by WHO clinical algorithms, wasting (unintentional loss >10% of total body weight (TBW)), oral/esophageal candidiasis, chronic diarrhea, *Pneumocystis jirovecii* pneumonia, cryotococcal meningitis, *Toxoplasma gondii* infection, and Kaposi's sarcoma were extracted from patient charts.

### Objective

The objective of this analysis was twofold: (1) to identify the proportion of individuals who experience CD4 cell counts ≤200 cells/mm^3^ and CD4 counts ≤500 cells/mm^3^ after 12 and 30 months of suppressive antiretroviral therapy, respectively, and (2) to identify factors that are associated with the change in CD4 cell counts over time in the South African patient population.

### Statistical analysis

Confidence intervals for restoration rates were estimated using methods for exact binomial confidence intervals. Baseline patient characteristics were compared using Wilcoxon rank sum and Fisher's exact tests. Multivariate analysis using logistic regression was employed to assess the predictors of CD4 cell restoration at months 12 and 30 (≥200 cells/mm^3^ after 12 and ≥500 cells/mm^3^ after 30 months). Analysis of repeated measures, using mixed models, was conducted to assess the overtime change in CD4 cell counts and identify the predictors of change in CD4 cell counts. The longitudinal modeling approach utilized in this study allows the intercept and the rate at which CD4 cell counts change over time to vary across participants. Moreover, the modeling approach does not require participants to have the same number of visits or measurements, and uses all available data instead of eliminating subjects with missing data, resulting in unbiased estimates of the model parameters when data are missing at random.

## Results

### Patient Characteristics

A total of 442 eligible patients who initiated antiretroviral therapy at McCord Hospital were included in the analyses. Sixty-one percent were female and the median age was 35.4 years (range 21–69 years) (table 1). Median CD4 cell count at baseline was 94.5 cells/mm^3^ (range 1.0–198 cells/mm^3^). Median BMI and Hb values at study entry were 22.7 kg/m^2^ (range 13.3–44.2) and 11.2 g/dl (range 4.7–19.6 g/dl), respectively (table 1). The proportion of patients with pulmonary TB, extra pulmonary TB and TB meningitis and were 28%, 14% and 1%, respectively. Overall, 43% of the participants had TB co-infection (pulmonary TB, TB meningitis or extra pulmonary TB) at study entry (table 1), Twenty-four percent of the patients presented with chronic diarrhea, while 31% and 36% had unintentional loss >10% of total body weight (TBW) and/or oral candidiasis, respectively. *Pneumocystis jirovecii* pneumonia, cryotococcal meningitis, *Toxoplasma gondii* infection and Kaposi's sarcoma were found in less than 5% of patients at study entry. The majority (81%) of patients initiated HAART on a combination regimen, including d4T, 3TC and Efavirenz (table 1). During the 30 months of follow-up, 64% of patients switched treatment regimens for toxicity at least once, primarily for neuropathy and lipodystrophy/lipoatrophy, while 16% switched regimens twice or more.

**Table 1:** CD4 count outcomes at 12 and 30 months based on patient demographic and pre-HAART characteristics.

Variables	CD4 counts at 12 months (N = 428)				CD4 counts at 30 months (N = 427)		
	<200		>200		<500	>500	
	Med(min-max)	Med(min-max)		P-value	Med(min-max)		P-value
**Age**	35.4 (20.8–68.9)	37(22–64)	35(21–69)	0.003	36(21–69)	34(22–58)	0.007
**Baseline CD4**	94.5 (1–198)	55(1–198)	115(1–198)	<0.0001	91(1–198)	109(3–197)	0.05
**Hemoglobin**	11.2 (4.7–19.6)	11(5–20)	11(6–17)	0.76	11(5–20)	11(5–16)	0.95

		<200	>200		<500	>500	
	N (%)	N (%)	N (%)	P-value	N (%)	N (%)	P-value
**Gender**				0.54			0.37
**Female**	269 (61)	95 (58)	163 (62)		209 (59)	49 (65)	
**Male**	173 (39)	68 (42)	102 (38)		143 (41)	26 (35)	
**BMI**				0.33			0.54
**<18.5**	52 (13)	21 (14)	29 (12)		42 (13)	9 (12)	
**18.5–25**	225 (54)	90 (58)	129 (53)		188 (56)	33 (50)	
**≥25**	135 (33)	44 (29)	87 (36)		103 (31)	25 (38)	
**TB infection**				0.61			0.19
**No**	245 (57)	93 (58)	145 (56)		200 (58)	36 (49)	
**Yes**	187 (43)	66 (42)	116 (44)		146 (42)	37 (51)	
**Wasting (>10% loss of TBW)**				0.16			0.49
**No**	297 (69)	102 (64)	185 (71)		235 (68)	53 (73)	
**Yes**	135 (31)	57 (36)	76 (29)		111 (32)	20 (27)	
**Candidiasis**				0.29			0.35
**No**	277 (64)	96 (60)	172 (66)		224 (65)	43 (59)	
**Yes**	155 (36)	63 (40)	89 (34)		122 (35)	30 (41)	
**Chronic Diarrhea**				0.25			0.46
**No**	328 (76)	115 (72)	202 (77)		265 (77)	53 (73)	
**Yes**	104 (24)	44 (28)	59 (23)		81 (23)	20 (27)	
**Treatment at study entry**				0.44			0.74
**D4T/3TC/EFV**	360 (81)	137 (84)	214 (81)		289 (82)	60 (80)	
**Other**	82 (19)	26 (16)	51 (19)		63 (18)	15 (20)	
**ART switch at 12 month**				0.59			-
**No**	307 (69)	115 (71)	179 (68)		-	-	
**Yes**	135 (31)	48 (29)	86 (32)		-	-	
**ART switch at 30 month**				-			0.23
**No**	158 (36)	-	-		131 (37)	22 (29)	
**Yes**	284 (64)	-	-		221 (63)	53 (71)	

### Failure to achieve a normal CD4+ T-cell count after 12 and 30 months of suppressive antiretroviral therapy

**Figure 1:**
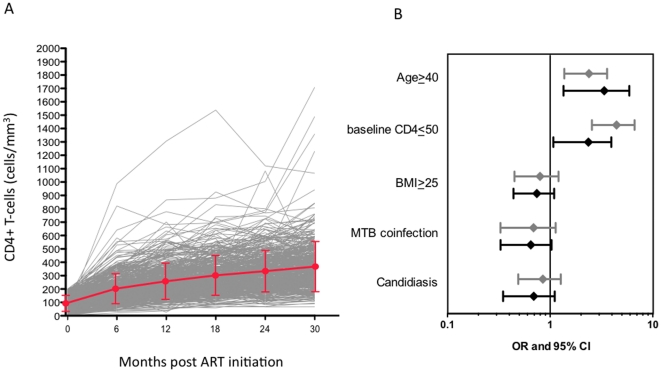
CD4+ T-cell count trajectories and baseline characteristics predicting failure of CD4 cell count recovery. **A**. CD4+ T-cell count trajectories for all 442 subjects over the course of 30 months. The red line reflects the median±SEM. **B**. OR and 95% confidence intervals (CI) for not achieving CD4 counts above 200 cells/mm^3^ at 12 months (grey) and 500 cells/mm^3^ at 30 months (black) depending on baseline characteristics. Shown are only factors with significant or close to significant effect in the multivariate model.

Among 442 eligible patients a total of 428 and 427 patients were available for CD4 analyses at 12 and 30 months respectively. A total of 163 patients (37%) among the 442 eligible patients, had a CD4 cell count <200 cells/mm^3^ at 12 month (figure 1A). Among those individuals, 113 patients eventually had a confirmed increase in their CD4 cell count to ≥200 cells/mm^3^ after 30 months, but the remainder 50 subjects had a persistently low CD4 cell count. Two patients remained below 100 cells/mm^3^ throughout the study follow-up. At 30 months, the majority of patients failed to achieve CD4 cell counts of ≥500 cells/mm^3^, with only 75 (17%) patients reaching this threshold (figure 1A).

### Predictors of CD4+ cell count restoration at 12 and 30 months

In order to identify the predictors of failure to achieve CD4 cell restoration ≥200 cells/mm^3^ at month 12 and ≥500 cells/mm^3^ at month 30, we performed a multivariate analysis, adjusting for multiple risk factors simultaneously. All variables that were associated with the outcomes at the 0.4 level in the univariate analyses were included in a list of candidate variables for the multivariate analyses. TB co-infection and BMI were included in the multivariate models regardless of their statistical significance in the univariate analyses. As shown before in HIV-1 clade B infection, age at treatment initiation was a strong predictor of CD4 cell restoration in our clade C infected cohort. Patients ≥40 years of age were significantly more likely to not achieve CD4 cell counts >200 cells/mm^3^ at 12 month (OR 2.22, 95% CI: 1.37–3.59; p = 0.001) and ≥500 cells/mm^3^ at 30 months (OR 2.83, 95% CI: 1.35–5.92; p = 0.0057) (figure 1B). Moreover, baseline CD4 cell count was another strong predictor of CD4 cell recovery. Individuals with CD4 cell counts below 50 cells/mm^3^ were more likely to not achieve CD4 cell counts above 200 cells/mm^3^ (OR = 4.12, 95% CI:2.55–6.64, p<0.0001) and 500 cells/mm^3^ (OR = 2.06, 95% CI:1.08–3.94, p = 0.0294) at 12 and 30 months, respectively (figure 1B). Other patient characteristics including gender, baseline BMI or hemoglobin were not associated with CD4 cell count restoration at 12 or 30 months. Despite a high prevalence of TB infection at baseline (43%), TB infection at time of HAART initiation did not undermine CD4 cell recovery. In contrast, individuals with TB at baseline tended to be at decreased risk for failure to recover to a CD4 cell counts below 500 cells/mm^3^ at 30 months (OR 0.58, 95% CI: 0.33–1.03; p = 0.065) despite significantly lower baseline CD4 cell counts in TB co-infected individuals versus TB negative subjects (median CD4 cell counts 72 vs 110 cells/mm^3^, p = 0.0003). Other frequent symptoms at study entry such as chronic diarrhea, unintentional weight loss >10% of TBW and oral/esophageal candidiasis were not associated with increased risk of CD4 cell counts <200 and <500 cells/mm^3^ at 12 and 30 months, respectively.

### CD4 cell count slopes based on pre-therapy characteristics

There was an overall increase in CD4 cell counts over time, with an average rate of increase of 8.66±0.44 cells/mm^3^ per month (p<0.0001). Although baseline CD4 cell counts were significantly associated with CD4 cell counts at the subsequent time points (p<0.0001), the rate of increase in CD4 cell numbers over time did not vary by baseline CD4 cell counts (p = 0.2744). Baseline age significantly predicted rate of change in CD4 cells over time (p = 0.0002) with lower rates for older (>40 yrs of age) patients (6.42 cells/mm^3^ per month) vs younger (<40 years of age) patients (8.66 cells/mm^3^ per month). Baseline TB co-infection status was not associated with CD4 cell counts (p = 0.5092). However, TB co-infected individuals showed a trend toward higher rates of increase in CD4 cells compared to non-TB patients (9.55 cells/mm^3^ versus 8.66 cells/mm^3^ per month, p = 0.1097). Gender, BMI status, switch of therapy regimens and treatment regimen had no effect on the overtime change in CD4 cell counts.

## Discussion

These data show that despite adequate virological response 37% and 83% of patients on antiretroviral therapy failed to restore CD4 cell counts to greater than 200 cells/mm^3^ after 12 months and greater than 500 cells/mm^3^ at 30 months, in a South African cohort under routine program conditions. We also demonstrate that South African HIV-infected patients initiating antiretroviral treatment at very depressed CD4 cell counts (<50 cells/mm^3^) or initiating HAART above the age of 40 years are less likely to achieve optimal CD4 cell restoration. These results are important because it has been shown in another South African cohort that total time spent at a CD4 cell count below 200 cells/mm^3^ is linked to increased morbidity and mortality [2]. These data provide further evidence that HIV-infected patients, eligible for HAART in South Africa, should be actively identified earlier in the course of disease, and at younger age.

Poor CD4 cell restoration is a consistent problem across diverse HIV-infected cohorts examined to date. The number of virologically suppressed persons not reaching CD4 cell count goals at 12 and 30 months in this cohort was comparable to reports from the United States, and Europe [11], [13], [14]. Although the link between low baseline CD4 cell counts <50cells/mm^3^ and failure to restore CD4 cell count with HAART has been previously noted, evidence has mainly been derived from European cohorts [7], [9], [11], [15], [16]. The link between age greater than 40 years and failure to achieve brisk CD4 cell count recovery has not previously been demonstrated in sub-Saharan Africa [9], [14].

As other studies have defined immunologic non-response, as not achieving CD4 cell counts ≥350 after at least a 2-year ART exposure we expanded our analysis to include the 350 CD4 cell count cut-off after 30 month of HAART. Age and baseline CD4 cell counts remained strong predictors of failed CD4 cell recovery (data not shown), while male gender became significantly associated with failure to restore CD4 cell counts (data not shown). This data is consistent with prior data linking male gender to unfavorable CD4 cell count outcome [17].

Interesting, nearly half of our study population presented with TB co-infection defined by positive AFB smear, radiographical evidence or high clinical suspicion at ART initiation. Although HIV/TB co-infected individuals had significantly lower CD4 cell counts at baseline compared to TB negative individuals, they showed significantly improved CD4 cell restorations ≥350 at 30 month (data not shown), with a trend towards more subjects reaching CD4 cell counts even ≥500 cells at 30 months. One possible explanation is that TB co-infection results in additional CD4 cell count suppression, which resolves with treatment of both infections [18].

Underlying mechanisms for poor CD4 cell restoration are not yet clear. However a recent report, among persons with declining CD4 cell count in the United States despite suppressive HAART, noted biopsy evidence of lymphoid tissue collagen deposition [19]. It is not known if lymphoid tissue fibrosis is also responsible for poor CD4 cell reconstitution in South African patients but does raise important concerns about the reversibility of the underlying process once underway.

The study had several strengths. We included a relatively large sample of patients (n = 442) observed under routine program conditions with minimal exclusion criteria. Patients were of Zulu/Xhosa background and represent a sample that differs significantly in gender, age, CD4 cell count nadir and co-infection from most US and European cohorts used in previously published analysis on CD4 cell restoration [7], [12], [13], [14], [15], [16], [20], [21], [22]. The majority of our patients were young females, with low CD4 cell nadir (below 100 cells/mm^3^) and high prevalence of TB co-infection at presentation, consistent with the population profile most affected in Sub-Saharan Africa [23]. Because the majority of our patients (81%) initiated a regimen containing d4T, 3TC and EFV, we were able to estimate CD4 cell recovery independent of potential effect of different ART regimens. During the course of the study, 64% of patients switched to another regimen, mostly for toxicity reasons but we found that changing regimen had no impact on CD4 cell restoration in accord with prior findings [8], [15]. Study weaknesses include the use of CD4 cell count alone as a proxy for immune restoration, absence of histological evidence to explain potential mechanisms for failure of CD4 cell recovery and limited information on clinical consequences of poor CD4 cell count restoration after the 30 month follow-up such as rates of opportunistic infections and mortality.

In summary we demonstrate here that a significant proportion of persons initiating HAART under routine conditions in South Africa fail to restore CD4 cell count despite adequate virologic response. Important risk factors are low CD4 cell count nadir and increased age at HAART initiation consistent with previously published data from industrialized countries. These data suggest that greater efforts are needed to identify and treat HAART-eligible patients prior to severe CD4 cell decline or achievement of advanced age.

## References

[1] Boulle A, Van Cutsem G, Hilderbrand K, Cragg C, Abrahams M (2010). Seven-year experience of a primary care antiretroviral treatment programme in Khayelitsha, South Africa.. Aids.

[2] Lawn SD, Little F, Bekker LG, Kaplan R, Campbel E (2009). Changing mortality risk associated with CD4 cell response to antiretroviral therapy in South Africa.. Aids.

[3] Nglazi MD, Lawn SD, Kaplan R, Kranzer K, Orrell C (2011). Changes in programmatic outcomes during 7 years of scale-up at a community-based antiretroviral treatment service in South Africa.. J Acquir Immune Defic Syndr.

[4] Sanne IM, Westreich D, Macphail AP, Rubel D, Majuba P (2009). Long term outcomes of antiretroviral therapy in a large HIV/AIDS care clinic in urban South Africa: a prospective cohort study.. J Int AIDS Soc.

[5] Barnighausen T, Tanser F, Gqwede Z, Mbizana C, Herbst K (2008). High HIV incidence in a community with high HIV prevalence in rural South Africa: findings from a prospective population-based study.. Aids.

[6] Battegay M, Nuesch R, Hirschel B, Kaufmann GR (2006). Immunological recovery and antiretroviral therapy in HIV-1 infection.. Lancet Infect Dis.

[7] Egger M, May M, Chene G, Phillips AN, Ledergerber B (2002). Prognosis of HIV-1-infected patients starting highly active antiretroviral therapy: a collaborative analysis of prospective studies.. Lancet.

[8] Gutierrez F, Padilla S, Masia M, Iribarren JA, Moreno S (2006). Clinical outcome of HIV-infected patients with sustained virologic response to antiretroviral therapy: long-term follow-up of a multicenter cohort.. PLoS ONE.

[9] Hughes MD, Daniels MJ, Fischl MA, Kim S, Schooley RT (1998). CD4 cell count as a surrogate endpoint in HIV clinical trials: a meta-analysis of studies of the AIDS Clinical Trials Group.. Aids.

[10] Hunt PW, Deeks SG, Rodriguez B, Valdez H, Shade SB (2003). Continued CD4 cell count increases in HIV-infected adults experiencing 4 years of viral suppression on antiretroviral therapy.. Aids.

[11] Kaufmann GR, Furrer H, Ledergerber B, Perrin L, Opravil M (2005). Characteristics, determinants, and clinical relevance of CD4 T cell recovery to <500 cells/microL in HIV type 1-infected individuals receiving potent antiretroviral therapy.. Clin Infect Dis.

[12] Mellors JW, Munoz A, Giorgi JV, Margolick JB, Tassoni CJ (1997). Plasma viral load and CD4+ lymphocytes as prognostic markers of HIV-1 infection.. Ann Intern Med.

[13] Florence E, Lundgren J, Dreezen C, Fisher M, Kirk O (2003). Factors associated with a reduced CD4 lymphocyte count response to HAART despite full viral suppression in the EuroSIDA study.. HIV Med.

[14] Moore RD, Keruly JC (2007). CD4+ cell count 6 years after commencement of highly active antiretroviral therapy in persons with sustained virologic suppression.. Clin Infect Dis.

[15] Khanna N, Opravil M, Furrer H, Cavassini M, Vernazza P (2008). CD4+ T cell count recovery in HIV type 1-infected patients is independent of class of antiretroviral therapy.. Clin Infect Dis.

[16] Mocroft A, Phillips AN, Gatell J, Ledergerber B, Fisher M (2007). Normalisation of CD4 counts in patients with HIV-1 infection and maximum virological suppression who are taking combination antiretroviral therapy: an observational cohort study.. Lancet.

[17] Collazos J, Asensi V, Carton JA (2007). Sex differences in the clinical, immunological and virological parameters of HIV-infected patients treated with HAART.. Aids.

[18] Wanchu A, Kuttiatt VS, Sharma A, Singh S, Varma S (2010). CD4 cell count recovery in HIV/TB co-infected patients versus TB uninfected HIV patients.. Indian J Pathol Microbiol.

[19] Nies-Kraske E, Schacker TW, Condoluci D, Orenstein J, Brenchley J (2009). Evaluation of the pathogenesis of decreasing CD4(+) T cell counts in human immunodeficiency virus type 1-infected patients receiving successfully suppressive antiretroviral therapy.. J Infect Dis.

[20] Chene G, Sterne JA, May M, Costagliola D, Ledergerber B (2003). Prognostic importance of initial response in HIV-1 infected patients starting potent antiretroviral therapy: analysis of prospective studies.. Lancet.

[21] Kaufmann GR, Perrin L, Pantaleo G, Opravil M, Furrer H (2003). CD4 T-lymphocyte recovery in individuals with advanced HIV-1 infection receiving potent antiretroviral therapy for 4 years: the Swiss HIV Cohort Study.. Arch Intern Med.

[22] Patterson K, Napravnik S, Eron J, Keruly J, Moore R (2007). Effects of age and sex on immunological and virological responses to initial highly active antiretroviral therapy.. HIV Med.

[23] UNAIDS (2008). Country report South Africa: Progress Report On Declaration Of Commitment On HIV And AIDS 2008.. http://www.unaids.org/en/dataanalysis/monitoringcountryprogress/2010progressreportssubmittedbycountries/2008progressreportssubmittedbycountries/south_africa_2008_county_progress_report_en.pdf.

